# Predictors of high sensitivity cardiac troponin T in chronic kidney disease patients: a cross-sectional study in the chronic renal insufficiency cohort (CRIC)

**DOI:** 10.1186/1471-2369-14-229

**Published:** 2013-10-22

**Authors:** Ruth F Dubin, Yongmei Li, Jiang He, Bernard G Jaar, Radhakrishna Kallem, James P Lash, Gail Makos, Sylvia E Rosas, Elsayed Z Soliman, Ray R Townsend, Wei Yang, Alan S Go, Martin Keane, Christopher deFilippi, Rakesh Mishra, Myles Wolf, Michael G Shlipak

**Affiliations:** 1 San Francisco VA Medical Center, University of California San Francisco, 4150 Clement Street, Box 111A1, San Francisco, CA 94121, USA; 2 Tulane University School of Public Health and Tropical Medicine, New Orleans, LA, USA; 3 Johns Hopkins University, Baltimore, MD, USA; 4 Perelman School of Medicine at University of Pennsylvania, Philadelphia, PA, USA; 5 University of Illinois at Chicago, Chicago, IL, USA; 6 St. John’s Health System, Detroit, MI, USA; 7 Epidemiological Cardiology Research Center (EPICARE), Department of Epidemiology, and Department of Internal Medicine, Section on Cardiology, Wake Forest School of Medicine, Winston-Salem, NC, USA; 8 University of Pennsylvania Scientific and Data Coordinating Center, Philadelphia, PA, USA; 9 Kaiser Permanente Northern California, Division of Research, Oakland, CA, USA; 10 University of Maryland Medical Center, Baltimore, USA; 11 University of Miami Miller School of Medicine, Miami, USA

**Keywords:** Troponin T, Chronic kidney disease, Cardiovascular disease

## Abstract

**Background:**

Cardiac troponin T is independently associated with cardiovascular events and mortality in patients with chronic kidney disease (CKD). Serum levels of high sensitivity cardiac troponin T (hs-TnT) reflect subclinical myocardial injury in ambulatory patients. We sought to determine the distribution and predictors of hs-TnT in CKD patients without overt cardiovascular disease (CVD).

**Methods:**

We studied 2464 participants within the multi-ethnic Chronic Renal Insufficiency Cohort (CRIC) who did not have self-reported CVD. We considered renal and non-renal factors as potential determinants of hs-TnT, including demographics, comorbidities, left ventricular (LV) mass, serologic factors, estimated glomerular filtration rate (eGFR) and albumin to creatinine ratio.

**Results:**

Hs-TnT was detectable in 81% of subjects, and the median (IQR) hs-TnT was 9.4 pg/ml (4.3-18.3). Analysis was performed using Tobit regression, adjusting for renal and non-renal factors. After adjustment, lower eGFR was associated with higher expected hs-TnT; participants with eGFR < 30 ml/min/1.73 m^2^ had 3-fold higher expected hs-TnT compared to subjects with eGFR > 60. Older age, male gender, black race, LV mass, diabetes and higher blood pressure all had strong, independent associations with higher expected hs-TnT.

**Conclusions:**

Knowledge of the determinants of hs-TnT in this cohort may guide further research on the pathology of heart disease in patients with CKD and help to stratify sub-groups of CKD patients at higher cardiovascular risk.

## Background

Studies have shown that lower estimated glomerular filtration (eGFR) and higher albumin to creatine ratio (ACR) are strong, independent risk factors for incident heart failure [1-3]. However, how chronic kidney disease (CKD) leads to heart failure is not fully understood; specifically, we lack information on which biological mediators of CKD initiate myocardial injury and whether certain subgroups of patients with CKD are more susceptible to myocardial injury. High sensitivity cardiac troponin T (hs-TnT) independently predicts cardiovascular mortality in populations with or without cardiac disease [4-6], and predicts cardiovascular events [7,8] and all-cause mortality [9] in patients with CKD and end-stage renal disease [10]. Since the highly sensitive assay detects much lower levels of myocardial injury than prior assays, it may be useful for studying the earliest stages of heart disease in subjects with CKD. Our group has shown that hs-TnT is independently associated with left ventricular hypertrophy (LVH) in participants of the CRIC cohort who do not have self-reported cardiovascular disease (CVD) [11]. Additional culprits in the CKD milieu thought to contribute to the development of CVD include inflammation, anemia, and deranged mineral metabolism. Whether these factors are associated with subclinical myocardial injury (as measured by hs-TnT) in a CKD cohort without overt CVD has not been previously studied.

We sought to examine renal and non-renal predictors of subclinical myocardial injury using hs-TnT in subjects without self-reported CVD in the Chronic Renal Insufficiency Cohort (CRIC). First, we hypothesized that lower estimated glomerular filtration rate (eGFR) and higher urine albumin-creatinine ratio (ACR) would be independently associated with higher hs-TnT concentration. Second, we hypothesized that elevated fibroblast growth factor 23 (FGF-23) concentrations, hyperphosphatemia, and anemia would be associated with higher hs-TnT independently of eGFR.

## Methods

The Chronic Renal Insufficiency Cohort (CRIC) Study was designed to investigate risk factors for progression of CKD, cardiovascular disease and overall mortality in persons with CKD. Participants were recruited between June 2003 and March 2007 at seven centers (Ann Arbor, Michigan; Baltimore, Maryland; Chicago, Illinois; Cleveland, Ohio; New Orleans, Louisiana; Philadelphia, Pennsylvania; and Oakland, California). Investigators recruited 3939 racially and ethnically diverse individuals between the ages of 21 to 74 years with eGFR between 20 and 70 ml/min/1.73 m^2^ by simplified MDRD equation [12]. Exclusion criteria were as follows: polycystic kidney disease, use of immunosuppression within the last 6 months, institutionalization, inability to consent, enrollment in other studies, pregnancy, New York Heart Association class III to IV heart failure, HIV, cirrhosis, myeloma, renal cancer, recent chemotherapy, organ transplant, or dialysis treatment within the last month [13]. The Institutional Review Board at each study site approved the protocol and participants gave written, informed consent. For this analysis, participants with known self-reported cardiovascular disease, peripheral vascular disease, or heart failure were excluded.

Kidney function was measured using cystatin-based eGFR (eGFRcys) and creatinine-based eGFR using the MDRD equation (eGFRcr). Compared to creatinine, cystatin has been shown to be a better marker of kidney function at higher eGFR [14], and cystatin has a stronger association with cardiovascular outcomes than creatinine-based eGFR (eGFRcr) [15,16]. In CRIC, eGFRcys has a wider distribution of values than eGFRcr because enrollment was based upon a fixed range of eGFR [17]. Samples for cystatin were processed using a Siemens BNII nephelometer at the CRIC central laboratory, with a coefficient of variation (CV) of 4.9%. Values were corrected for drift over time by standardization to calibrator lot 51 and reagent lot 40. Cystatin-c based eGFR was calculated by the CKD-EPI equation [18], and eGFR was categorized as < 30, 30–44, 45–59, and ≥ 60 ml/min/1.73 m^2^. Urine samples were collected for spot albumin-creatinine ratios (ACR), and ACR was categorized as < 30, 30–299, 300–999, and ≥ 1000 mg/g.

Information on demographics and clinical history was obtained by self-report on questionnaires administered at the baseline visit. Diabetes was defined as documented medical history, current or previous use of diabetic medications, or elevated fasting blood glucose. Blood pressure was averaged over three measurements performed in a standardized fashion in a seated position at rest using a calibrated sphygmomanometer. Echocardiograms were performed at one year after enrollment and were read at the core echocardiography laboratory (University of Pennsylvania) using guidelines of the American Society of Echocardiography [19]. Left ventricular mass index was derived by the area-length method, indexed to height^2.7^[19]. Hs-TnT was measured at Rapid Response Laboratories, University of Maryland Medical Center with the Roche Elecsys immunoassay (Roche Diagnostics, Indianapolis, Indiana), which has an analytical range of 3 to 10,000 ng/L, and a coefficient of variation of 9% at 13.5 ng/L (the 99th percentile in a healthy reference group) [20].

### Statistical analysis

Of 2505 subjects without CVD, 2464 had hs-TnT measurements, and of these 474 (19%) were undetectable (< 3 pg/ml). We first categorized hs-TnT as undetectable (< 3 pg/ml), Tertile 1 (3–8.6 pg/ml), Tertile II (8.7-17 pg/ml), and Tertile III (17–739 pg/ml). We compared characteristics of participants in these four categories using ANOVA or Kruskal-Wallis tests for continuous variables and Chi-squared tests for categorical variables.

We next performed multivariable regression analysis in order to identify factors with independent associations with hs-TnT. Because such a large proportion of hs-TnT values were undetectable, we used tobit regression, which is designed to handle left-censored values. Undetectable values were assigned the level 1.5 pg/ml. Because hs-TnT is right-skewed, the outcome was log-transformed for analysis. Coefficients were then exponentiated to yield the estimated multiplicative increase attributable to each factor; e.g., an exponentiated coefficient of 1.3 represents a 30% higher hs-TnT in the exposed compared with the unexposed. Initially, the regression model included all candidate covariates with p-value < 0.05 by univariate analysis. Model 1 was built using backwards stepwise selection until only covariates significant at p < 0.05 remained. Model 2 was built by adding LV mass and ejection fraction to Model 1. All analyses were performed using STATA 11 (StataCorp LP, College Station, TX).

## Results

**Figure 1:**
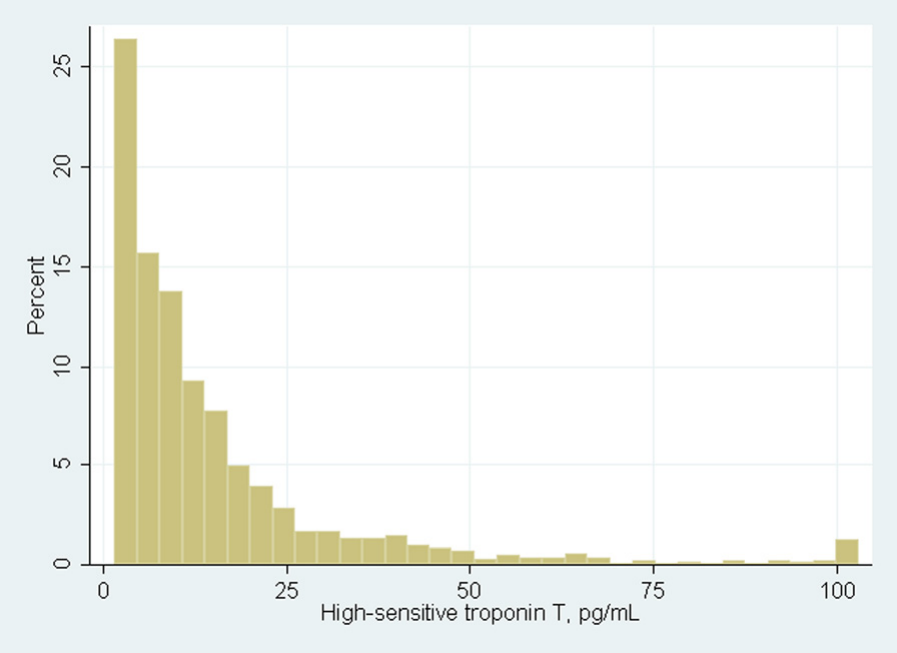
**Distribution of high sensitivity troponin t among chronic renal insufficiency cohort participants without self**-**reported cardiovascular disease.** Legend: Hs-TnT was detectable in 81% of subjects; median (IQR) of hs-TnT was 9.4 pg/ml (4.3-18.3). All hs-TnT values greater than 100 pg/ml were truncated in one bar at 100.

In this subgroup of CRIC study participants without CVD, hs-TnT was detectable in 81% of subjects, and median (IQR) of hs-TnT was 9.4 pg/ml (4.3-18.3) (Figure 1). Increased age, male gender, black race and Hispanic ethnicity were associated with higher TnT, as were diabetes and hypertension. Subjects with higher hs-TnT had lower LDL, lower HDL, higher tryglycerides, lower hemoglobin, higher phosphorus, higher FGF-23, lower eGFRcys and higher ACR (Table 1).

**Table 1:** Characteristics of chronic renal insufficiency cohort participants with high sensitivity troponin t measurements

	**Undetectable**	**Tertile I**	**Tertile II**	**Tertile III**	**P**
	**(hs**-**TnT < 3)**	**(3.00**-**8.63)**	**(8.67**-**17.03)**	**(17.05**-**738.7)**	
	**(n=474)**	**(n=666)**	**(n=661)**	**(n=663)**	
**Hs**-TnT (pg/ml)*	1.5 (1.5-1.5)	5.7 (4.6-7.1)	11.9 (10.1-14.2)	30.0 (21.4-45.3)	< 0.001
**Age**	51.5 (11.8)	55.7 (11.8)	60.3 (10.7)	59.2 (11.1)	< 0.001
**Female**	75.5	53.8	42.7	29.1	< 0.001
**Race**					< 0.001
**Non**-**Hispanic White**	53.2	49.9	45.1	29.1	
**Non**-**Hispanic Black**	33.8	35.6	38.0	45.4	
**Hispanic**	8.4	9.0	13.9	21.4	
**Other**	4.6	5.6	3.0	4.1	
**Diabetes**	14.8	29.1	45.1	68.3	< 0.001
**Hypertension**	68.6	82.1	91.4	94.7	< 0.001
**Current smoking**	13.1	10.4	11.8	11.0	0.526
**SBP (mmHg) †**	124.1 (16.9)	128.3 (17.3)	132.9 (19.5)	140.5 (20.9)	< 0.001
**DBP (mmHg) ‡**	75.1 (12.1)	75.5 (11.9)	74.3 (12.0)	76.8 (11.8)	0.010
**BMI (kg/m**^**2**^**) §**	30.6 (8.5)	31.2 (7.9)	32.3 (8.1)	32.5 (7.5)	< 0.001
**HDL (mg/dL) ||**	54.1 (17.7)	50.9 (15.2)	49.1 (16.2)	46.4 (15.4)	< 0.001
**LDL (mg/dL)**	108.9 (30.4)	107.7 (34.1)	102.4 (34.7)	98.7 (36.3)	< 0.001
**Triglycerides (mg/dL)**	144.2 (114.1)	155.2 (115.1)	149.3 (91.1)	161.3 (108.0)	< 0.001
**Total cholesterol (mg/dL)**	193.7 (37.6)	192.3 (41.4)	184.3 (44.8)	182.0 (45.6)	< 0.001
**Serum albumin (g/dL)**	4.2 (0.4)	4.1 (0.4)	4.1 (0.4)	3.9 (0.5)	< 0.001
**hsCRP (mg/L) #**	4.9 (8.3)	4.9 (8.8)	5.4 (8.7)	5.7 (9.8)	0.015
**Hemoglobin (g/dL)**	13.2 (1.5)	13.2 (1.8)	13.0 (1.8)	12.2 (1.8)	< 0.001
**Phosphate (mg/dL)**	3.6 (0.6)	3.6 (0.5)	3.6 (0.6)	3.9 (0.7)	< 0.001
**FGF**-23 (RU/ml)**	104.7 (73.8-160.2)	114.6 (81.3-182.5)	137.4 (93.3-208.8)	171.7 (113.9-293.0)	< 0.001
**eGFRcys †† (ml/min/1.73 m**^**2**^**)**	66.1 (22.7)	55.9 (19.7)	49.5 (16.3)	40.9 (16.2)	< 0.001
**ACR (μg/mg) ψ**	11.2 (5.0-86.9)	21.3 (5.4-201.7)	30.5 (7.9-279.7)	289.7 (31.4-1393.2)	< 0.001
**LV mass (g/m**^**2.7**^**) Ω**	43.8 (10.2)	46.1 (10.4)	50.3 (12.8)	55.4 (13.8)	< 0.001
**Ejection fraction (%)**	55.7 (6.3)	55.9 (6.4)	55.8 (7.4)	54.3 (7.3)	< 0.001

Mean (SD) or median (IQR) are reported for continuous variables and percentage for categorical variables. P values are obtained using ANOVA or Kruskal-Wallis tests for continuous variables and Chi-squared tests for categorical variables. * High sensitivity troponin T. † Systolic blood pressure. ‡ Diastolic blood pressure. § Body mass index. || High density lipoprotein. Low density lipoprotein. # High sensitivity c-reactive protein. ** Fibroblast growth factor 23. †† Cystatin-based estimated glomerular filtration rate. ψ Albumin to creatinine ratio. Ω Left ventricular.

**Figure 2:**
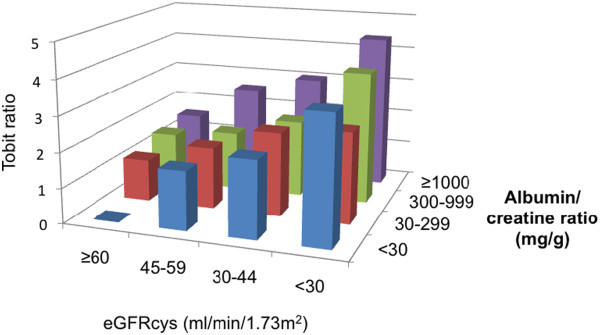
**Joint associations of categories of estimated glomerular filtration rate and albumin to creatinine ratio with high sensitivity troponin t in adjusted tobit regression analyses.** Legend: After multivariate adjustment, lower eGFR and higher albuminuria were associated with higher levels of hs-TnT. Final Tobit model adjusted for: age (per SD), female, race, diabetes, SBP categories, albumin (per SD), LV mass, ejection fraction, eGFRcys, ACR, and phosphate and FGF-23 (per SD). SD: standard deviation. SBP: systolic blood pressure. LV: left ventricular. eGFRcys: cystatin- based estimated glomerular filtration rate. ACR: albumin to creatinine ratio. FGF-23: fibroblast growth factor 23.

After multivariate adjustment, the strongest renal predictor of hs-TnT was eGFRcys. All eGFRcys categories below the referent group of > 60 ml/min/1.73 m^2^ were associated with incrementally higher hs-TnT. Subjects with eGFRcys < 30 ml/min/1.73 m^2^ had almost a 3-fold higher hs-TnT than the referent group with eGFRcys > 60 ml/min/1.73 m^2^. ACR did not show a linear association with hs-TnT; only those subjects with ACR ≥ 1000 mg/g had higher hs-TnT compared to those with ACR < 30 mg/g. Associations of phosphate and FGF-23 with hs-TnT were significantly attenuated by adjustment, and hemoglobin lost significance with adjustment (compared to referent category of hemoglobin > 14, hemoglobin <=11 had 16% higher hs-TnT, p=0.07) (Table 2). We found an additive effect between eGFRcys and ACR when we further categorized subjects by both of these factors. After multivariate adjustment, those in the lowest eGFR/highest ACR category (eGFRcys< 30 ml/min/1.73 m^2^ and ACR ≥ 1000) had more than a 4-fold increase in hs-TnT compared to the referent group (eGFRcys> 60 ml/min/1.73 m^2^ and ACR< 30) (Figure 2).

**Table 2:** Associations between renal factors and high sensitivity troponin t

		**Unadjusted**		**Model 1**		**Model 2**	
	N*	**Tobit ratio (95% CI)**	**P**	**Tobit ratio (95% CI)**	**P**	**Tobit ratio (95% CI)**	**P**
eGFRcys* (ml/min/1.73 m^**2**^**)**							
≥ 60	757	Reference	-	Reference		Reference	-
45-59	684	2.11 (1.86-2.41)	< 0.001	1.51 (1.35-1.69)	< 0.001	1.50 (1.34-1.67)	< 0.001
30-44	705	3.02 (2.66-3.44)	< 0.001	1.97 (1.74-2.22)	< 0.001	1.96 (1.74-2.21)	< 0.001
< 30	311	5.27 (4.47-6.21)	< 0.001	2.90 (2.47-3.41)	< 0.001	2.83 (2.41-3.33)	< 0.001
**ACR (μg/mg) †**							
< 30	1115	Reference	-	Reference		Reference	-
30-299	582	1.58 (1.39-1.80)	< 0.001	1.08 (0.98-1.20)	0.130	1.06 (0.96-1.19)	0.214
300-999	338	1.94 (1.66-2.28)	< 0.001	1.09 (0.96-1.25)	0.191	1.08 (0.95-1.23)	0.238
≥ 1000	325	3.61 (3.08-4.23)	< 0.001	1.56 (1.35-1.81)	< 0.001	1.53 (1.32-1.77)	< 0.001
**Phosphate (mg/dL)**							
< 3	265	Reference	-	Reference		Reference	-
3-3.9	1427	1.08 (0.90-1.28)	0.419	1.02 (0.90-1.17)	0.739	1.02 (0.90-1.17)	0.711
4-4.9	646	1.59 (1.31-1.93)	< 0.001	1.06 (0.91-1.23)	0.445	1.06 (0.92-1.24)	0.412
≥ 5	81	3.51 (2.52-4.90)	< 0.001	1.35 (1.04-1.76)	0.023	1.39 (1.08-1.81)	0.012
**FGF**-**23 ‡**							
Quartile I	606	Reference		Reference		Reference	
Quartile II	606	1.45 (1.25-1.68)	< 0.001	1.07 (0.96-1.20)	0.230	1.08 (0.96-1.21)	0.206
Quartile III	606	2.11 (1.82-2.45)	< 0.001	1.20 (1.06-1.35)	0.004	1.17 (1.04-1.33)	0.011
Quartile IV	606	2.62 (2.26-3.04)	< 0.001	1.22 (1.06-1.40)	0.004	1.17 (1.02-1.34)	0.021

*N’s are # observations among participants with TnT measurements, with 7 observation excluded.

Model 1 adjusted for: age (per SD), female, race, diabetes, SBP categories, albumin (per SD), eGFR_CysC, ACR, and phosphate and FGF-23. Model 2 adjusted for: Model 1 + LV mass, ejection fraction.

eGFRcys=cystatin-based estimated glomerular filtration rate. † albumin to creatinine ratio. ‡ fibroblast growth factor 23.

Several non-renal covariates remained strong predictors of hs-TnT after multivariable adjustment. Increased age was associated with higher hs-TnT, and hs-TnT was substantially lower in women compared to men. Compared to non-Hispanic whites, non-Hispanic blacks had significantly higher hs-TnT; although Hispanic participants had higher TnT levels, the association was not significant. Higher LV mass index was associated with higher hs-TnT across all categories of LV mass index; lower ejection fraction was associated with higher hs-TnT mainly in the lowest category (≤ 35%). Diabetes, elevated SBP and lower serum albumin were independently associated with higher hs-TnT (Table 3). After adjustment, the following markers were not associated with higher hs-TnT (B coefficient per SD (95% CI)): hsCRP 0.98 (0.94-1.02), p=0.386; LDL 1.01 (0.97-1.06), p=0.510; HDL 1.04 (0.99-1.09), p=0.126; triglycerides 0.99 (0.95-1.03), p=0.597. The associations of renal and non-renal covariates were consistent across categories of renal function. Substitution of eGFRcr for eGFRcys did not change our findings.

**Table 3:** **Associations between non**-**renal factors and high sensitivity troponin t**

		**Unadjusted**		**Model 1**		**Model 2**	
	N*	**Tobit ratio (95% CI)**	** P**	**Tobit ratio (95% CI)**	** P**	**Tobit ratio (95% CI)**	** P**
**Age (per SD)**	2,457	1.39 (1.32-1.46)	< 0.001	1.31 (1.25-1.37)	< 0.001	1.31 (1.25-1.37)	< 0.001
**Female**	1,190	0.41 (0.37-0.46)	< 0.001	0.38 (0.35-0.41)	< 0.001	0.39 (0.36-0.42)	< 0.001
**Race**							
Non-Hisp white	1,075	Reference	-	Reference	-	Reference	-
Non-Hisp Black	946	1.51 (1.34-1.70)	< 0.001	1.31 (1.20-1.43)	< 0.001	1.26 (1.16-1.38)	< 0.001
Hispanic	330	2.09 (1.77-2.47)	< 0.001	1.06 (0.93-1.21)	0.398	1.02 (0.89-1.16)	0.768
Other	106	1.10 (0.84-1.44)	0.492	1.03 (0.84-1.25)	0.807	1.02 (0.84-1.24)	0.854
**Diabetes**	1,009	2.96 (2.68-3.28)	< 0.001	1.87 (1.71-2.04)	< 0.001	1.84 (1.69-2.00)	< 0.001
**Albumin (per SD)**	2,437	0.72 (0.68-0.76)	< 0.001	0.92 (0.88-0.96)	0.001	0.93 (0.89-0.98)	0.003
SBP (mmHg)*							
< 120	408	Reference	-	Reference	-	Reference	-
120-129	324	1.32 (1.09-1.61)	0.005	1.03 (0.89-1.19)	0.678	1.03 (0.89-1.19)	0.706
130-139	321	1.69 (1.39-2.05)	< 0.001	1.14 (0.98-1.32)	0.081	1.12 (0.97-1.29)	0.139
140-149	194	2.03 (1.62-2.55)	< 0.001	1.24 (1.05-1.47)	0.014	1.22 (1.03-1.44)	0.023
150-159	131	2.71 (2.09-3.51)	< 0.001	1.44 (1.19-1.76)	< 0.001	1.38 (1.14-1.69)	0.001
≥ 160	163	3.36 (2.64-4.27)	< 0.001	1.35 (1.12-1.63)	0.001	1.27 (1.06-1.53)	0.011
**LV mass (g/m**^**2.7**^**) †**							
< 40	474	Reference	-			Reference	-
40-59	1,165	1.94 (1.68-2.24)	< 0.001			1.17 (1.05-1.30)	0.006
60-79	284	3.89 (3.20-4.72)	< 0.001			1.39 (1.19-1.63)	< 0.001
80-99	41	3.77 (2.49-5.72)	< 0.001			1.47 (1.04-1.97)	0.018
≥ 100	10	4.85 (2.15-10.95)	< 0.001			1.87 (1.02-3.42)	0.043
**Ejection fraction (%)**							
> 50	1,845	Reference	-			Reference	-
46-50	197	1.12 (0.92-1.38)	0.253			1.13 (0.98-1.31)	0.087
36-45	130	1.33 (1.04-1.69)	0.023			1.13 (0.95-1.34)	0.184
≤ 35	22	1.93 (1.09-3.41)	0.024			1.64 (1.09-2.48)	0.018

*N’s are # observations among participants with TnT measurements, with 7 observation excluded.

Model 1 adjusted for: age (per SD), female, race, diabetes, SBP categories, albumin (per SD), eGFR_CysC, ACR, and phosphate and FGF-23. Model 2 adjusted for: Model 1 + LV Mass, ejection fraction. * Systolic blood pressure. † Left ventricular.

## Discussion

In this study of participants without CVD selected from the CRIC cohort, over 80% had detectable levels of hs-TnT. Lower eGFR, higher LV mass, increased age, male gender, black race, diabetes and higher blood pressure were independently associated with higher hs-TnT. Interpreting these associations may help us understand which subjects with CKD are most susceptible to developing coronary disease or heart failure.

The median hs-TnT in our cohort (9 pg/ml) was comparable to subjects with stable coronary artery disease (5–6 pg/ml [21]) and heart failure (13–17 pg/ml [22]). This degree of chronic, low-level troponin release has been attributed to various mechanisms in addition to macrovascular atherosclerotic disease. Left ventricular hypertrophy (LVH) is common in this population, and is associated with higher levels of hs-TnT [11]. Chronic kidney disease may lead to myocardial injury via endothelial dysfunction and microvascular disease caused by elevated levels of asymmetric dimethylarginine (ADMA) [23] or mediators of oxidative stress [24]. Alternatively, the association between lower eGFR and higher TnT could be in part due to reduced renal clearance of troponin T. Troponin T fragments small enough to be cleared by the kidneys have been found in serum of patients with ESRD [25]. However, we also show that LV mass is associated with hs-TnT independently of eGFR; furthermore, 5% of persons with eGFR< 30 and 10% with eGFR between 30–45 had undetectable hs-TnT, demonstrating that impaired clearance is unlikely to be solely responsible for detectable levels of hs-TnT.

Similar to the Dallas Heart Study in which hs-TnT was studied in a community-based cohort [4] we found that older age, male gender, black race and diabetes were independently associated with elevated hs-TnT. While it seems intuitive that older age and diabetes would be associated with microvascular injury and thus lead to higher hs-TnT, the explanation for male gender and black race is less obvious. Male gender and black race are associated with higher LV mass index [26-28], but in our study these associations persisted after adjustment for LV mass index. African Americans have been found to have a higher risk of endothelial dysfunction [29,30], and this may explain the higher levels of hs-TnT among them in our cohort.

Prior studies have shown FGF-23 to have strong, independent associations with structural cardiovascular disease including LVH [31], coronary artery disease [32], and carotid intima-media thickness [33]; cardiovascular events [34]; and overall mortality [13,35] in cohorts with prevalent CVD and all stages of CKD [13]. One role of FGF-23 in cardiovascular disease is via induction of left ventricular hypertrophy; FGF-23 injected into mice at concentrations of 800 RU/ml resulted in LVH [36]. In addition, the growth factor was associated with myocardial injury (hs-TnT) in a CKD cohort, an association that was only mildly attenuated by adjustment for LVH [37]. In contrast, we observed only a weak association of FGF-23 with hs-TnT levels with or without adjustment for LV mass index. The fact that we excluded CRIC participants with history of CVD may account for these differences. While FGF-23 does not appear to be a predominant factor for subclinical myocardial injury in CRIC subjects without CVD, it may yet prove crucial in the development of heart failure in this cohort via induction of LVH.

Strengths of our study include the large study cohort, broad range of eGFRcys and ethnic diversity represented by CRIC. The high percentage of participants with detectable hs-TnT increased our power to detect cross-sectional associations. There are also several important limitations. Subjects with known cardiovascular disease were excluded based on history, not left heart catheterization; some subjects with undetected coronary artery disease may have been included. Since our analysis is cross-sectional, we cannot assume a causal relationship between factors associated with hs-TnT and hs-TnT or incident CVD. We cannot separate the relative importance of hs-TnT production vs. clearance as determinants of serum concentrations.

## Conclusions

We conclude that in the absence of CVD, the most important renal predictor of higher hs-TnT in the CRIC cohort is lower eGFRcys. Non-renal predictors include age, male gender, black race, diabetes, and higher systolic blood pressure. Further studies will evaluate whether hs-TnT is associated with adverse outcomes in CRIC.

## Abbreviations

CRIC: Chronic renal insufficiency cohort; CKD: Chronic kidney disease; CVD: Cardiovascular disease; Hs-TnT: High sensitivity troponin T; SBP: Systolic blood pressure; DBP: Diastolic blood pressure; BMI: Body mass index; LDL: Low density lipoprotein; hsCRP: High sensitive C-reactive protein; FGF-23: Fibroblast growth factor 23; eGFRcys: Cystatin-based estimated glomerular filtration; eGFRcr: Creatinine-based estimated glomerular filtration; MDRD: Modification of diet in renal disease; ACR: Albumin to creatinine ratio; LV: Left ventricular; LVH: Left ventricular hypertrophy; SD: Standard deviation; IQR: Interquartile range; ADMA: Asymmetric dimethylarginine.

## Competing interests

The authors declare that they have no competing interests.

## Authors’ contributions

RD participated in the formulation of hypotheses, statistical analyses and drafted the paper. YL conducted statistical analyses. MS is responsible for study conception and design as well as supervising manuscript completion. The remaining authors were all involved in critically revising the manuscript content and format. All authors have given final approval of the version to be published.

## Pre-publication history

The pre-publication history for this paper can be accessed here:

http://www.biomedcentral.com/1471-2369/14/229/prepub
